# Indication Criteria for Total Hip Arthroplasty in Patients with Hip Osteoarthritis—Recommendations from a German Consensus Initiative

**DOI:** 10.3390/medicina58050574

**Published:** 2022-04-22

**Authors:** Cornelia Lützner, Stefanie Deckert, Klaus-Peter Günther, Anne Elisabeth Postler, Jörg Lützner, Jochen Schmitt, David Limb, Toni Lange

**Affiliations:** 1 University Center of Orthopedics, Trauma and Plastic Surgery, University Hospital Carl Gustav Carus Dresden, Technische Universität Dresden, Fetscherstr. 74, 01307 Dresden, Germany; cornelia.luetzner@ukdd.de (C.L.); anne.postler@ukdd.de (A.E.P.); joerg.luetzner@ukdd.de (J.L.); 2 Center for Evidence-Based Healthcare, University Hospital Carl Gustav Carus and Carl Gustav Carus Faculty of Medicine Carl Gustav Carus, Technische Universität Dresden, Fetscherstr. 74, 01307 Dresden, Germany; stefanie.deckert@ukdd.de (S.D.); jochen.schmitt@ukdd.de (J.S.); toni.lange@ukdd.de (T.L.); 3 Chapel Allerton Hospital, Leeds Teaching Hospitals Trust, Harehills Lane, Leeds LS7 4SA, UK; d.limb@leeds.ac.uk

**Keywords:** total hip arthroplasty, guideline, indication criteria, shared decision making, illness-related burden

## Abstract

*Background:* Osteoarthritis of the hip (hip OA) is a leading cause of pain and disability in elderly people. If non-surgical therapies become ineffective, patients may consider total hip arthroplasty (THA). The biggest challenge in recommending a THA is identifying patients for whom the benefits of this procedure outweigh the potential risks. The aim of this initiative was to develop a clinical practice guideline with accompanying algorithm to guide consultations on THA, supported by a pocket-sized checklist. *Methods:* The initiative “Evidence- and consensus-based indication criteria for total hip replacement (EKIT-Hip)” used a stepwise approach, starting with an inauguration workshop, where a multidisciplinary German stakeholder panel from various scientific societies agreed on the working process. A Project Coordinating Group (PCG) was formed, and it performed a comprehensive systematic literature search of guidelines and systematic reviews related to the indication criteria for THA, as well as factors influencing outcomes. Based on best-available evidence, preliminary recommendations were formulated by the PCG and discussed with the stakeholder panel during a consensus meeting. In addition, the panel was asked to assess the feasibility of an extracted algorithm and to approve a final checklist. *Results:* In total, 31 recommendations were approved by 29 representatives of 23 societies. These were used to underpin an algorithm (EKIT-Algorithm), which indicates the minimum requirements for a THA (confirmed diagnosis of hip OA, present and documented individual burden of illness, ineffectiveness of non-surgical therapies, and absence of any contraindications). Once these criteria are fulfilled, further considerations should encompass the medical implications of modifiable risk factors and patients’ individual treatment goals, as discussed during shared decision making. The subsequently developed checklist (EKIT-Checklist) lists relevant criteria for decision making. *Conclusions:* Adherence to the EKIT-Algorithm, conveniently accessed via the EKIT-Checklist, should improve the standardization of decision making leading to a recommendation for THA. By applying minimum requirements and patient-related risk factors, as well as considering patients’ individual goals, it is possible to identify patients for whom the benefits of THA may exceed the potential risks.

## 1. Introduction

Osteoarthritis (OA) is one of the leading causes of pain and disability in elderly people [1]. Guideline recommendations for non-surgical treatment strategies applicable to OA of the hip (hip OA) are well established, for example, EULAR [2], OARSI [3], NICE [4], and DGOOC [5]. Given the degenerative nature of OA, there is currently no preventative treatment available [1,6]. Relief of symptoms and associated impairment are the main objectives of non-surgical therapy. If available conservative treatment strategies become ineffective and a patient develops end-stage hip OA, total hip arthroplasty (THA) may be considered. THA is a highly effective treatment option [1,7] and has become one of the most frequently performed routine surgical procedures in the past decades [8]. In Germany, 175,000 primary THA were performed in 2019 [9]. Regarding numbers of THA procedures, forecasts from various countries show a consistently rising demand [10,11,12,13,14,15,16] with significant implications for economic and human resources in the health system [16].

Nevertheless, THA is an elective and irreversible procedure. In addition, not all patients benefit equally with respect to improvement of pain, physical function, health-related quality of life, and long-term implant survival. Therefore, identification of patients for whom the benefits of this procedure are likely to exceed the potential risks becomes the most challenging part of the consultation, and a wise decision is essential for the appropriate utilization of health care resources.

Rates of THA performed vary substantially among and within countries [17,18,19], which might partly be explained by the absence of standardized indication criteria during consultation and shared decision making processes. In 2019, a survey among 147 German orthopedic surgeons about the criteria for and against THA surgery, as well as alternative treatment options, was performed [20]. Participants rated pain (99%), limitation of movement (99%), and impaired walking distance (97%), as well as the individual burden of illness (97%) to be the most important criteria for recommending THA to their patients. Prescription of analgesics and physical therapy, as well as a lack of their effectiveness, were considered by 97% and 96%, respectively, as suitable criteria for THA. A total of 87% identified radiological changes at a minimum Kellgren & Lawrence grade III as a suitable threshold [20].

However, to our knowledge, there is currently no generally accepted clinical practice guideline, neither internationally nor in Germany, that exclusively addresses the indication criteria for THA [21]. Therefore, the German initiative “Evidence- and consensus-based indication criteria for total hip arthroplasty (EKIT-Hip)” was established. The aim of this activity was to compile a clinical practice guideline to guide consultations related to THA in hip OA [22]. In general, guidelines are systematically developed statements that reflect the current state of knowledge and provide assessment of the benefits and harms of alternative treatment options. Based on a systematic review of the available evidence and the consensus of a multidisciplinary German stakeholder panel, this guideline should cover the sequence of the indication process—from diagnosis to shared decision making—and provide universal and practical recommendations. This is a novel and unique approach and a summary report of the recommendations has recently been published in German and English languages [23,24].

The aim of this article is to describe the consensus process, elaborate in detail on the indications as well as the contraindications identified as suitable criteria for THA, and to propose a pocket-sized checklist that can support consultations and shared decision making in routine care. After careful assessment and, if necessary, adaption to national requirements, these indication criteria for THA might also be applicable in other countries outside the German healthcare system or considered as a starting point for the development of a nation’s own bespoke criteria.

## 2. Materials and Methods

### 2.1. Inauguration Workshop

As a first step in the EKIT-Hip initiative, a Project Coordinating Group (PCG) was established, consisting of members with appropriate clinical and methodological expertise. This group organized an inauguration workshop on 21 June 2017 in Dresden, Germany, where the methodological approach was discussed with a multidisciplinary panel (EKIT-Consensus-Panel). Members of this Panel were nominated by relevant scientific societies, health insurers, as well as patient organizations. Subsequently, the EKIT-Consensus-Panel defined the evidence needed for the consensus-building process and, correspondingly, the strategies for evidence retrieval and the designated consensus procedure. The following levels of consensus were agreed, which are defined by the Association of the Scientific Medical Societies in Germany (AWMF):

- No consensus, less than 50% agreement of the EKIT-Consensus-Panel;
- Weak consensus, 50% to 74% agreement of the EKIT-Consensus-Panel;
- Consensus, 75% to 94% agreement of the EKIT-Consensus-Panel;
- Strong consensus, 95% or more agreement of the EKIT-Consensus-Panel [25].

In addition, it was agreed that the PCG would draft preliminary recommendations based on the evidence retrieved and present them for discussion and voting at the consensus meeting.

### 2.2. Evidence Retrieval, Synthesis, and Derivation of Preliminary Recommendations

In preparation for the literature search, the PCG defined an outline algorithm for decision making in THA (EKIT-Algorithm) that consisted of the following six steps which, it is suggested, should be followed sequentially during consultation and shared decision making:

(1) Confirmation of diagnosis;
(2) Identification of a patient’s individual burden of illness;
(3) Checking alternative treatment options;
(4) Identification of contraindications;
(5) Identification of modifiable risk factors;
(6) Shared decision making.

These predefined six steps in the algorithm were approved by the EKIT-Consensus-Panel and served as a basis for consecutive systematic literature searches. The best available evidence was identified by two systematic literature reviews. The PCG searched for national/international clinical practice guidelines and systematic reviews dealing with hip OA and THA. In addition, two reviews on outcome measurement instruments and shared decision making were performed to gain further evidence.

The initial search for clinical practice guidelines was performed across 20 databases (Supplementary Figure S1) between August and September 2019 and subsequently updated (last update in January 2020). Only evidence-based guidelines were included, which contained a consensus process, published in German or English languages after 31 December 2010, and addressing physicians. All identified guidelines were screened by one reviewer, and all guidelines identified for potential inclusion were verified by a second reviewer.

The initial search for systematic reviews was conducted in MEDLINE (via Pubmed) and EMBASE (via Ovid) initially in August 2019 and subsequently updated (last update in August 2020). Overall, a sensitive literature search was applied by using the search terms “hip OA”, “Total Hip Arthroplasty” and employing the search filter for systematic reviews and meta-analyses, as proposed by SIGN [26]. Systematic reviews published in German or English languages were included. Two reviewers screened the titles and abstracts and subsequently the full text, considering the six predefined topics of the EKIT-Algorithm.

Essential information was extracted from the identified guidelines, and systematic reviews were extracted into evidence tables by one reviewer and approved by a second reviewer. Quality appraisal was performed independently by two reviewers using guidelines from the German *instrument for methodological guideline appraisal* (DELBI tool, domain 3 and 6) [27] and for systematic reviews with *a measurement tool for the assessment of multiple systematic reviews* (AMSTAR 2) [28]. According to all findings, preliminary recommendations on the predefined topics and related explanatory texts were developed by the PCG. Finally, the drafted information was provided to the EKIT-Consensus-Panel, along with voting documents, four weeks prior to the EKIT-Consensus-Meeting.

### 2.3. EKIT-Consensus-Meeting

Prior to the consensus meeting, conflicts of interest (COI) of the representatives were evaluated. All representatives agreed that participants with COI (defined as obtaining fees for expert opinion, participation in company advisory boards, and/or receiving royalties in the context of THA implant development) should not participate in voting on eight preliminary recommendations (Supplementary Table S1). An experienced independent moderator, delegated from the AWMF, assisted in the consensus process. At the beginning of the consensus meeting, the voting procedure was agreed. Subsequently, each preliminary recommendation and its explanatory text were shortly introduced, followed by the discussion and voting process. In the case of a disagreement, recommendations were rephrased. Voting categories were “agree”, “disagree” and “abstain”. Voting and counting were performed in real time and the results were presented immediately after each vote. Then, each agreed recommendation was compiled into the final clinical practice guideline.

### 2.4. Development of a Checklist of Indication Criteria

To facilitate the transfer of the identified criteria for decision making on THA into routine care, (1) a graphical illustration of the EKIT-Algorithm, as well as (2) a pocket-sized checklist were developed by the PCG. The checklist was modeled based on the proposed checklist of a previous guideline initiative addressing the indication criteria for arthroplasty in knee OA [29]. The feasibility of both resources was evaluated by orthopedic members of the PCG and finally approved by the EKIT-Consensus-Panel.

## 3. Results

### 3.1. Evidence Retrieval

**Figure 1:**
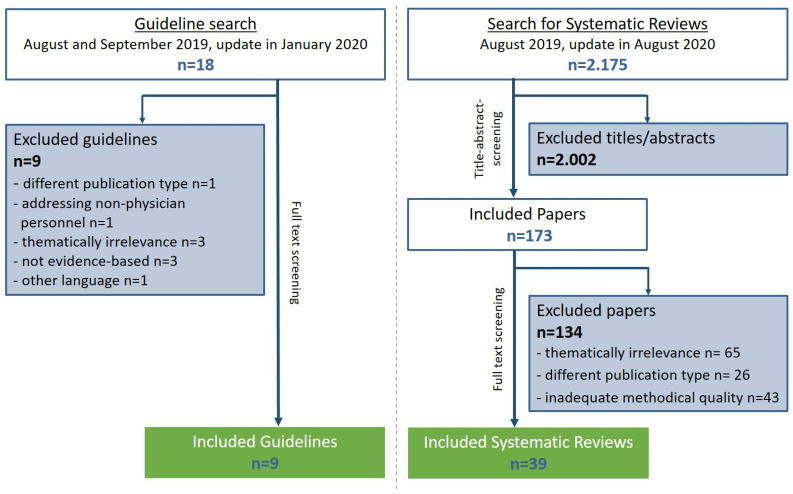
Flow chart of the systematic literature search for clinical practice guidelines and the systematic reviews in the context of hip OA and THA.

Among the 18 guidelines identified during the systematic literature search, 9 guidelines were classified as methodologically adequate (according to the DELBI criteria) and were further considered for integration into guideline recommendations. Additionally, one relevant German consensus-based guideline on the topic of hip OA was included [5]. Furthermore, out of 2175 initial hits, 39 relevant and methodologically adequate (according to AMSTAR 2) systematic reviews (33 with meta-analysis) were included (Figure 1). A summary of the results of the evidence retrieval has been published elsewhere [24]. Evidence tables can be found at the website of the AWMF [22].

### 3.2. Consensus Voting

The EKIT-Consensus-Meeting took place as a hybrid (online and face-to-face) meeting on 21 September 2020. In total, 29 representatives of 23 societies were involved. The PCG drafted 31 preliminary recommendations, of which 18 recommendations were rephrased before the final voting. Supplement Table S1 shows the final 31 recommendations regarding consultations on the suitability of THA in patients with hip OA.

### 3.3. Development of a Checklist of Indication Criteria for THA in Hip OA

**Figure 2:**
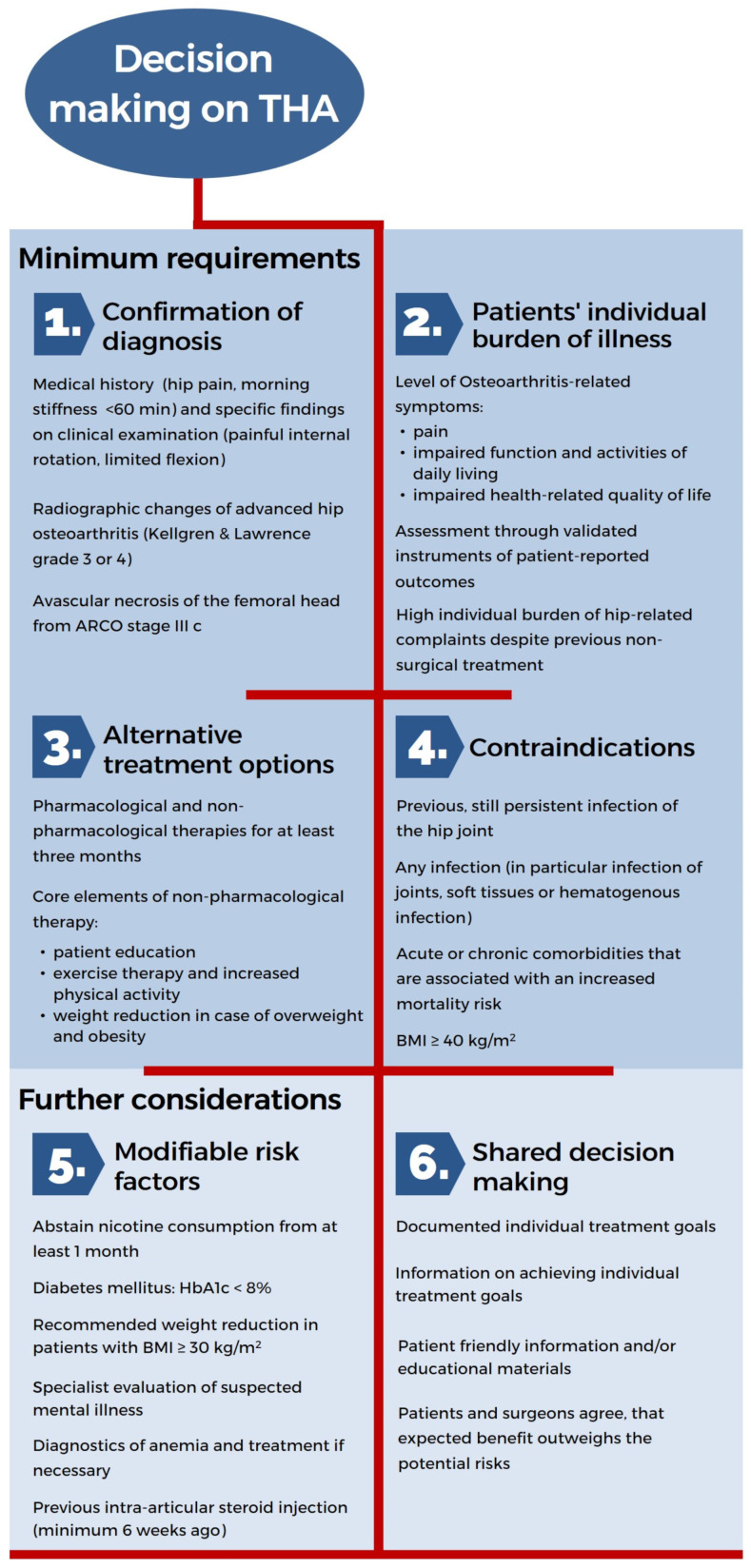
EKIT-Algorithm for decision making on THA.

The EKIT-Algorithm was underpinned by the final approved guideline recommendations and summarized accordingly (Figure 2). The key result of this process was the establishment of minimum requirements governing consultations on THA. Thus, orthopedic surgeons should:

(1) Ensure the diagnosis of hip OA via clinical examination and radiological signs;
(2) Assess the patient’s individual burden of illness regarding hip-related complaints and health-related quality of life using validated measurement instruments;
(3) Verify the use of alternative non-surgical therapies and their effectiveness during the last three months;
(4) Confirm the absence of any contraindications.

Once these four minimum requirements for recommending a THA are fulfilled, further considerations should encompass:

(5) Review of the medical implications regarding modifiable risk factors;
(6) The patient’s individual treatment goals and the probability of their achievement.

**Figure 3:**
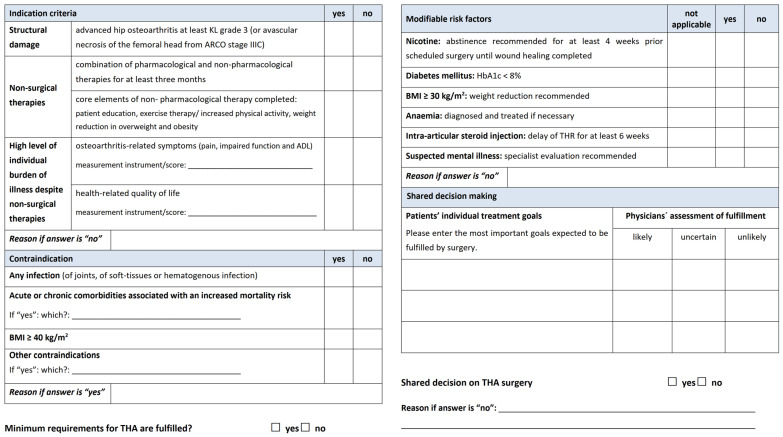
EKIT-Checklist of indication and contraindication criteria for decision making on THA.

Consequently, this process led to the development of the EKIT-Checklist, which facilitates documentation of the consultation on THA, including the status of minimum requirements, modifiable risk factors, patients’ individual goals, and the result of shared decision making on suitability of THA (Figure 3, full size version Supplementary Figure S2). Based on the feasibility check of orthopedic PCG members, the EKIT-Consensus-Panel approved both resources.

## 4. Discussion

### 4.1. Main Findings

Shared decision making between patients and surgeons on the suitability of THA is a complex and interactive process that should be based on objective criteria, including clinical and radiological findings along with subjective criteria, including the individual burden of illness and a patient’s expectations. Due to the irreversibility of THA, the appropriate timing of surgery should be decided mutually, when the surgeon and patient coming together to agree that the expected benefits outweigh the potential risks. In order to fulfill these requirements, a profound and practical guide to set standards governing consultations on THA is required.

The proposed clinical practice guideline is based on the best-available evidence and a broad consensus of German scientific societies, health insurers, and patient representatives. It provides 31 recommendations for decision making on THA, including minimum requirements that should be met before recommending a THA and a checklist for easy transfer of these requirements into routine care. The overall aim is to ensure a high quality of consultation on THA throughout Germany. Therefore, the underlying evidence, as well as the consensus-based recommendations, should be regularly reviewed and kept up to date. To the best of our knowledge, an algorithm for decision making on THA has not previously been developed and approved in this way by a broad range of stakeholders with relevant expertise in Germany.

### 4.2. International Perspective

Currently available guideline recommendations in the context of hip OA and THA do not refer to the criteria for the indications for surgery in such a detailed manner [21]. THA can be considered in patients with end-stage OA [30], who experience joint symptoms with substantial impact on their quality of life [4], after non-surgical options have been failed [4,30], and before there is prolonged and established functional limitation and severe pain [4]. Nicotine abuse, poorly controlled comorbidities, obesity, and mental health disorders are identified as the main risk factors for inferior outcomes and complications [30,31]. Therefore, dealing with modifiable risk factors was addressed in the EKIT-Hip initiative, resulting in specific guideline recommendations. New developments in this area are rare. In a recent update of the NICE guideline “Osteoarthritis: Care and Management” [4] and a novel NICE guideline “Joint Replacement (Primary): Hip, Knee and Shoulder” [32], no explicit recommendations on the indications or contraindication criteria were included. In summary, there is no comparable guideline published that focuses on the consultation itself, making specific recommendations for or against THA [21].

### 4.3. Clinical Considerations and Practical Implications

While considering THA for a patient, the recommended minimum requirements should be assessed and met before a decision is made for or against the procedure. If all of them are fulfilled, a decision in favor of a THA is reasonable, and modifying risk factors as well as considering a patient’s individual treatment goals can be addressed to achieve a properly informed, shared decision.

In specific cases, such as when a patient’s individual burden of illness is overwhelmingly high and no effective alternative treatment option is available, the decision to offer a THA may be reasonable even though the minimum requirements are not met. In contrast, recommending a THA is not justified if the possible harms due to identified risk factors exceed the potential benefits of the procedure to the patient, despite the fact that minimum requirements have been met, especially, if there are compliance issues or no scope for optimization.

In conclusion, when applying the EKIT-Algorithm, surgeons still need to weigh the benefits and risks for the patient individually.

### 4.4. Strengths and Limitations

This study has two main strengths. Comprehensive literature reviews were conducted to obtain the best available evidence and to inform the EKIT-Consensus-Panel, which was a multidisciplinary panel that represented a wide range of relevant stakeholders involved in the care of patients with hip OA. However, it needs to be acknowledged that global generalization of the results comes with limitations. Overall, transferability to other healthcare systems is limited by the unique German economic imperative principles, which form the basis of resource-oriented care in Germany. These imply that health care provision—including elective surgery—must be sufficient, appropriate, and economical. Moreover, the evidence considered in the consensus process is based mainly on expert judgements or indirect evidence (e.g., studies on outcome predictors) because controlled studies that have examined outcomes when comparing multiple indication criteria sets are lacking.

### 4.5. Implications for Further Research

With this paper, we aim to disseminate the indication criteria we have developed and make them available to the international community. The EKIT-Algorithm and EKIT-Checklist we have provided might be a resource to guide further and future guideline projects in elective surgery. In Germany, nationwide implementation, as well as evaluation of guideline adherence, are intended. Similar to the existing approaches by the EKIT-Knee initiative [33], the integration of this EKIT-Checklist into a digital decision aid could be initiated with a focus on feasibility, effectiveness, and the potential benefits for surgeons and patients. Regardless of the chosen format (analogue or digital), the provided EKIT-Checklist might also be a resource for a standard reporting format in clinical registries, such as the German Arthroplasty Registry (EPRD) or within hospital information systems. Given the increasing trends of total joint arthroplasty worldwide [10,11,12,13,14,15], high quality consultations are essential to identify those patients who are most likely to benefit from THA. This implies that guideline recommendations should be based on up-to-date evidence (living guidelines).

## 5. Conclusions

In Germany, a broad consensus amongst relevant stakeholders has been reached on indications and contraindications criteria for THA to be considered in consultations with patients seeking further treatment for hip OA. The proposed algorithm includes minimum requirements for surgery, optimization of risk factors, and consideration of patients’ individual treatment goals during shared decision making. The EKIT-Checklist is easy to apply in routine care and can be used for documentation as well as justification of the decision on surgery. These proposed instruments are based on systematically developed recommendations, which reflect the current state of knowledge and expertise. Adherence to the recommended algorithm for decision making in THA enhances standardized consultations, fostering higher quality of health care, and may ultimately lead to higher patient satisfaction.

## Data Availability

Comprehensive tables of the identified evidence can be found at the website of the AWMF (Leitlinienreport) [22].

## Supplementary Materials

The following supporting information can be downloaded at: https://www.mdpi.com/article/10.3390/medicina58050574/s1, Figure S1: List of databases for guideline search, Figure S2: EKIT-Checklist of indication and contraindication criteria for decision making on THA, Table S1: Recommendations regarding consultations on the suitability of THA (with voting results).
