# Supplementary material for: Indication Criteria for Total Hip Arthroplasty in Patients with Hip Osteoarthritis—Recommendations from a German Consensus Initiative
Source: Medicina (Kaunas). 2022 Apr 22;58(5):574. doi: 10.3390/medicina58050574 (PMC9146188; doi:10.3390/medicina58050574)
Supplement: Supplementary file 1 [file medicina-58-00574-s001.zip › medicina-1668100-supplementary.pdf]

Supplement Table S1: Recommendations regarding consultations on the suitability of THA (with voting results)

	**Recommendations**	**Agree**	**Disagree**	**Abstain**	**Consensus**
1.	Confirmation of diagnosis				
1.1	The diagnosis of hip osteoarthritis (hip OA) should be clinically confirmed by an appropriate medical history (hip pain, morning stiffness < 60 min) and specific findings on clinical examination (painful internal rotation and limited flexion of the hip).	25 (96%)	1 (4%)	0 (0%)	Strong consensus
1.2	Relevant differential diagnoses of hip OA should be excluded prior considering total hip arthroplasty (THA). This applies in particular to younger patients, in whom joint-preserving surgery may still be possible.	26 (96%)	0 (0%)	1 (4%)	Strong consensus
1.3	Prior to recommending THA to the patient, conventional radiographs (anteroposterior pelvic view and lateral view of the affected hip) should be available, to confirm the diagnosis and to assess the extent of degenerative changes.	27 (100%)	0 (0%)	0 (0%)	Strong consensus
1.4	For decision making in relation to THA, additional imaging with MRI and/or CT is only necessary if there is a discrepancy between clinical and plain radiological findings.	27 (100%)	0 (0%)	0(0%)	Strong consensus
1.5	In case of uncertain diagnosis, an image guided intra-articular injection with local anaesthetic can be performed before THA is recommended.	27 (93%)	1 (3%)	1(3%)	Consensus
1.6	Before patients undergo THA, the surgeon should assess the severity of the hip OA by considering the medical history, clinical examination and review of radiographs.	29 (100%)	0 (0%)	0 (0%)	Strong consensus
1.7 *	THA should only be performed in cases with radiographic changes of advanced OA (Kellgren & Lawrence grade 3 or 4).	24 (96%)	0 (0%)	0 (0%)	Strong consensus
1.8	In avascular necrosis of the femoral head, THA should be considered from ARCO stage IIIC.	26 (93%)	0 (0%)	2 (7%)	Consensus
2.	Patients' individual burden of illness				
2.0	Prior to recommending THA, the patients' individual burden of illness should be assessed by the level of the following OA-related symptoms:<br>- pain<br>- impaired function and Activities of Daily Living (ADL)<br>- impaired health-related quality of life.	29 (100%)	0 (0%)	0 (0%)	Strong consensus
2.1	Validated instruments for the assessment of patient-reported outcomes should be used, to determine the individual burden of illness. For this purpose, disease-specific and generic measurement tools are available.	30 (100%)	0 (0%)	0 (0%)	Strong consensus
2.3 *	THA should be considered in patients reporting a high individual burden of hip-related complaints (pain, limited function and ADL) along with impaired health-related quality of life, despite previous non-surgical treatment.	25 (100%)	0 (0%)	0 (%)	Strong consensus

	Recommendations	Agree	Disagree	Abstain	Consensus
2.4	During the informed consent discussion, the risks and benefit of early versus delayed THA should be discussed with patients, taking into account their individual burden of symptoms	28 (100%)	0 (0%)	0 (0%)	Strong consensus
3.	Alternative treatment options				
3.1	Prior to considering THA, patients with symptomatic hip OA should first be treated with a combination of pharmacological and non-pharmacological therapies.	27 (96%)	0 (0%)	1 (4%)	Strong consensus
3.2	Prior to considering THA, patients should have undergone, or been recommended, at least the following core elements of non-pharmacological therapy:<br>- patient education (information, education and counselling on the disease),<br>- exercise therapy and increased physical activity,<br>- weight reduction in case of overweight and obesity.	25 (89%)	2 (7%)	1 (4%)	Consensus
3.3 *	THA should be considered if patients complain of a high individual burden of illness despite guideline-adherent treatment for at least three months.	21 (84%)	4 (16%)	0 (0%)	Consensus
4.	Contraindications				
4.1	Prior to THA in a joint with a history of infection, checks should be performed to identify persistence.	26 (93%)	0 (0%)	2 (7%)	Consensus
4.2	Prior to performing THA any infection (in particular infection of joints, soft tissues or hematogenous infection) should be treated and cured.	23 (85%)	1 (4%)	3 (11%)	Consensus
4.3	In cases with acute or chronic comorbidities that are associated with an increased mortality risk, surgeons should only recommend THA or advice on the timing of such surgery, after risk assessment has been provided by an anaesthesiologist and any relevant specialist for significant comorbidities.<br>A second opinion from an orthopaedic surgeon may be sought to confirm the recommendation.	27 (93%)	0 (0%)	2 (7%)	Consensus
4.4 *	Due to the substantially increased risk of complications, a particularly critical benefit-risk analysis of THA should be performed in patients with a BMI ≥40 kg/m^2.	23 (92%)	0 (0%)	2 (8%)	Consensus
5.	Modifiable risk factors				
5.1 *	In cases with modifiable risk factors, patients should be given the option to postpone THA in order to minimize these risks before surgery.	25 (100%)	0 (0%)	0 (%)	Strong consensus
5.1.1 *	Smokers should be encouraged to abstain from their nicotine consumption from at least 1 month prior to scheduled THA.	24 (100%)	0 (0%)	0 (0%)	Strong consensus
5.1.2 *	a. In patients with diabetes mellitus, blood glucose should be controlled as best as possible.<br><br>b. Target is a HbA1c value below 8%	a. 23 (96%)<br><br>b. 24 (96%)	a. 0 (0%)<br><br>b. 0 (0%)	a. 1 (4%)<br><br>b. 1 (4%)	Strong consensus
5.1.3 *	In patients with a BMI ≥ 30 kg/m^2, weight reduction prior to THA should be recommended.	22 (92%)	0 (0%)	2 (8%)	Consensus

	Recommendations	Agree	Disagree	Abstain	Consensus
5.1.4	Treatment of asymptomatic bacteriuria is not a prerequisite for performing THA.	25 (89%)	1 (4%)	2 (7%)	Consensus
5.1.5	If mental illness is suspected, patients should be advised to seek specialist evaluation before THA is performed.	25 (89%)	2 (7%)	1 (4%)	Consensus
5.1.6	Prior to THA, anaemia should be diagnosed and if necessary treated.	26 (90%)	0 (0%)	3 (10%)	Consensus
5.1.7	In cases who have undergone previous intra-articular steroid injection, THA should be delayed at least for 6 weeks or even for three months beyond the date of the last injection.	22 (88%)	0 (0%)	3 (12%)	Consensus
6.	Shared decision making				
6.1	Patients should be encouraged to express individual treatment goals. These goals should be documented during shared decision making.	26 (93%)	1 (4%)	1 (4%)	Consensus
6.2	Patients should be informed of the extent to which individual treatment goals are achievable by THA or using alternative therapeutic options.	29 (100%)	0 (0%)	0 (0%)	Strong consensus
6.3	Patient friendly information and/or educational materials should be used whilst obtaining informed consent.	29 (100%)	0 (0%)	0 (0%)	Strong consensus
6.4	As a result of shared-decision making for THA, patients and surgeons should agree, that the expected benefit of treatment outweighs the potential risks.	29 (100%)	0 (0%)	0 (0%)	Strong consensus

Notes: * In total 5 participants of the EKIT-Consensus-Panel were excluded from voting due to COI, fees for expert opinion, participation in company advisory boards and/or receiving royalties in the context of THA implant development.

Supplementary Figure S1: List of databases for guideline search.

Cross-disciplinary databases of guidelines:

- Arzneimittelkommission der deutschen Ärzteschaft (AkdÄ)
- Association of the Scientific Medical Societies in Germany (AWMF)
- Canadian Medical Association (CMA)
- Centre for Reviews and Dissemination Health Technology Appraisals database (CRD HTA)
- Dutch Guideline Database
- Finnish Medical Society Duodecim
- Guidelines International Network (G-I-N)
- Institute for Clinical Systems Improvement (ICSI)
- National Guideline Clearinghouse (NGC)
- National Institute for Health and Clinical Excellence (NICE)
- New Zealand Guidelines Group National Health and Medical Research Council (NHMRC)
- Scottish Intercollegiate Guidelines Network (SIGN)

Specific databases:

- American Academy of Orthopaedic Surgeons (AAOS)
- American Association of Hip and Knee Surgeons (AAHKS)
- American College of Rheumatology (ACR)
- British Orthopaedic Association (BOA)
- European League Against Rheumatism (EULAR)
- New Zealand Orthopaedic Association (NZOA)
- Osteoarthritis Research Society International (OARSI)
- Outcome Measures in Rheumatology (OMERACT)

Figure S2: EKIT-Checklist of indication and contraindication criteria for decision making on THA.

**Indication criteria**		yes	no
**Structural damage**	advanced hip osteoarthritis at least KL grade 3 (or avascular necrosis of the femoral head from ARCO stage IIIC)		
**Non-surgical therapies**	combination of pharmacological and non-pharmacological therapies for at least three months		
	core elements of non- pharmacological therapy completed: patient education, exercise therapy/ increased physical activity, weight reduction in overweight and obesity		
**High level of individual burden of illness despite non-surgical therapies**	osteoarthritis-related symptoms (pain, impaired function and ADL)<br>measurement instrument/score: ______________________________		
	health-related quality of life<br>measurement instrument/score: ______________________________		
Reason if answer is "no"			

**Contraindication**	yes	no
**Any infection** (of joints, of soft-tissues or hematogenous infection)		
**Acute or chronic comorbidities associated with an increased mortality risk**<br>If "yes": which?: ____		
**BMI ≥ 40 kg/m²**		
**Other contraindications**<br>If "yes": which?: ____		
Reason if answer is "yes"		

Minimum requirements for THA are fulfilled? ☐ yes ☐ no

Modifiable risk factors	not applicable	yes	no
**Nicotine:** abstinence recommended for at least 4 weeks prior scheduled surgery until wound healing completed			
**Diabetes mellitus:** HbA1c < 8%			
**BMI ≥ 30 kg/m²:** weight reduction recommended			
**Anaemia:** diagnosed and treated if necessary			
**Intra-articular steroid injection:** delay of THR for at least 6 weeks			
**Suspected mental illness:** specialist evaluation recommended			
Reason if answer is "no"			

**Shared decision making**			
**Patients' individual treatment goals** Please enter the most important goals expected to be fulfilled by surgery.	**Physicians´ assessment of fulfillment**		
	likely	uncertain	unlikely

Shared decision on THA surgery ☐ **yes** ☐ **no**

Reason if answer is "no": ____

____
